# Isoforms of Neuropilin-2 Denote Unique Tumor-Associated Macrophages in Breast Cancer

**DOI:** 10.3389/fimmu.2022.830169

**Published:** 2022-04-27

**Authors:** Rajeev Dhupar, Katherine E. Jones, Amy A. Powers, Seth H. Eisenberg, Kai Ding, Fangyuan Chen, Cecile Nasarre, Zhanpeng Cen, Yi-Nan Gong, Amanda C. LaRue, Elizabeth S. Yeh, James D. Luketich, Adrian V. Lee, Steffi Oesterreich, Michael T. Lotze, Robert M. Gemmill, Adam C. Soloff

**Affiliations:** ^1^ Department of Cardiothoracic Surgery, University of Pittsburgh School of Medicine, Pittsburgh, PA, United States; ^2^ Cancer Immunology and Immunotherapy Program, University of Pittsburgh Medical Center (UPMC) Hillman Cancer Center, Pittsburgh, PA, United States; ^3^ Surgical Services Division, VA Pittsburgh Healthcare System, Pittsburgh, PA, United States; ^4^ Women’s Cancer Research Center, UPMC Hillman Cancer Center, Magee Women’s Research Institute, Pittsburgh, PA, United States; ^5^ Division of Hematology, Department of Medicine, Medical University of South Carolina, Charleston, SC, United States; ^6^ Division of Oncology, Department of Medicine, Medical University of South Carolina, Charleston, SC, United States; ^7^ Hollings Cancer Center, Medical University of South Carolina, Charleston, SC, United States; ^8^ School of Medicine, Tsinghua University, Beijing, China; ^9^ Department of Immunology, University of Pittsburgh School of Medicine, Pittsburgh, PA, United States; ^10^ Department of Pathology and Laboratory Medicine, Medical University of South Carolina, Charleston, SC, United States; ^11^ Research Service, Ralph H. Johnson VA Health Care System, Charleston, SC, United States; ^12^ Department of Pharmacology and Toxicology, Indiana University School of Medicine, Simon Cancer Center, Indianapolis, IN, United States; ^13^ Department of Pharmacology and Chemical Biology, University of Pittsburgh, Pittsburgh, PA, United States; ^14^ Department of Surgery, University of Pittsburgh School of Medicine, Pittsburgh, PA, United States; ^15^ Department of Bioengineering, University of Pittsburgh School of Medicine, Pittsburgh, PA, United States

**Keywords:** neuropilin, tumor-associated macrophage, breast cancer, neuropilin-2, neuropilin isoforms, neuropilin-2a, neuropilin-2b

## Abstract

Tumor-associated macrophages (TAMs) exert profound influence over breast cancer progression, promoting immunosuppression, angiogenesis, and metastasis. Neuropilin-2 (NRP2), consisting of the NRP2a and NRP2b isoforms, is a co-receptor for heparin-binding growth factors including VEGF-C and Class 3 Semaphorins. Selective upregulation in response to environmental stimuli and independent signaling pathways endow the NRP2 isoforms with unique functionality, with NRP2b promoting increased Akt signaling *via* receptor tyrosine kinases including VEGFRs, MET, and PDGFR. Although NRP2 has been shown to regulate macrophage/TAM biology, the role of the individual NRP2a/NRP2b isoforms in TAMs has yet to be evaluated. Using transcriptional profiling and spectral flow cytometry, we show that NRP2 isoform expression was significantly higher in TAMs from murine mammary tumors. NRP2a/NRP2b levels in human breast cancer metastasis were dependent upon the anatomic location of the tumor and significantly correlated with TAM infiltration in both primary and metastatic breast cancers. We define distinct phenotypes of NRP2 isoform-expressing TAMs in mouse models of breast cancer and within malignant pleural effusions from breast cancer patients which were exclusive of neuropilin-1 expression. Genetic depletion of either NRP2 isoform in macrophages resulted in a dramatic reduction of LPS-induced IL-10 production, defects in phagosomal processing of apoptotic breast cancer cells, and increase in cancer cell migration following co-culture. By contrast, depletion of NRP2b, but not NRP2a, inhibited production of IL-6. These results suggest that NRP2 isoforms regulate both shared and unique functionality in macrophages and are associated with distinct TAM subsets in breast cancer.

## Introduction

Macrophages are essential components of the innate immune response clearing cellular debris *via* phagocytosis/efferocytosis, orchestrating the induction of adaptive immunity, and mediating the resolution of inflammation. To account for the dynamic needs of tissues/organs, macrophages are intimately attuned to environmental signals which endow functional polarization and epigenetic imprinting. Tumor-associated macrophages (TAMs) are frequently the most prevalent leukocyte in breast tumors and have emerged as an independent co-factor in breast cancer metastasis (1). Distinct niches within the tumor microenvironment (TME) may promote TAM-mediated angiogenesis, immunosuppression, and metastasis, or opposingly, endow TAMs with cytotoxic and inflammatory tumoricidal functionality.

Neuropilins (NRPs) are evolutionarily conserved transmembrane glycoprotein co-receptors with broad tissue distribution in adult vertebrates (2, 3). The NRP family consists of NRP1 as well as six transcript variants of NRP2 including NRP2a, NRP2b, and a secreted form generated by alterative splicing (NRP2a22, NRP2a17, NRP2a0, NRP2b5, NRP2b0, and S9NRP2) (4). NRPs interact with growth factors, Class 3 Semaphorins (Sema3), and integrins, act as alternative VEGF receptors, and enhance signaling from cognate receptors such as receptor tyrosine kinases (RTKs). Through interactions with plexins and signaling intermediates, NRPs regulate cytoskeletal dynamics and axonal guidance, as well as cell/tumor cell proliferation, angiogenesis, lymphangiogenesis, and survival, and can either promote or inhibit tumor growth depending on interacting ligands and signaling pathways (5). Whereas signaling of NRP1 and NRP2a is regulated by a carboxyl terminal PDZ binding motif and its association with PDZ-domain factors such as GIPC (RGS-GAIP-interacting protein), the cytoplasmic domain of NRP2b interacts with GS3Kβ mediating independent pathways (6, 7). Recently, we demonstrated in lung cancer cells that the pro-tumorigenic activity of NRP2 resides primarily in the NRP2b isoform, which is upregulated during TGFβ-induced epithelial-mesenchymal transition (EMT) and is associated with more advanced stage, increased metastasis, and resistance to tyrosine kinase inhibitors (8, 9). Mechanistically, signaling to Akt was promoted by NRP2b *via* recruitment of RTKs and GSK3β, a known PTEN inhibitor, whereas Akt was inhibited by NRP2a *via* recruitment of PTEN to rapidly extinguish Akt activity (9).

The role of NRPs in regulating immunity has been increasingly appreciated, but the contribution and importance of each member is poorly understood (10, 11). NRP1 recruits and subsequently arrests pro-tumorigenic TAMs in response to Sema3A-producing hypoxic tumor regions through plexinA1/plexinA4 signaling and VEGFR1 activation (12). By comparison, little is known about NRP2 in immunity and there are no reports examining NRP2 isoform expression in leukocytes. NRP2 is undetectable in monocytes but is expressed on tissue-resident macrophages as well as TAMs within mouse mammary tumors and lung cancer patients (13–16). NRP2 expression in macrophages has been associated with both inflammatory (“M1-like”) as well as tolerogenic (“M2-like”) functionality (13, 15, 17, 18). Genetic ablation of NRP2 in TAMs impaired clearance of apoptotic tumor cells, resulting in secondary necrosis and subsequent inflammation within tumors leading to increased CD8^+^ T cell and NK cell recruitment and delayed tumor growth (19). However, the pro- or anti-tumor functions of the isoforms NRP2a and NRP2b remain undefined.

We hypothesized that NRP2a and NRP2b play distinct roles in macrophage function and may be important in TAM-mediated metastatic events in breast cancer. Using murine cell lines and mammary tumor models along with analysis of human primary and metastatic breast cancers, we provide the first evidence of isoform-specific NRP2 expression and its consequences for macrophage function. We demonstrate that NRP2 isoform expression regulates macrophage cytokine production, phagocytosis/lysosomal processing, and the ability to promote tumor cell migration *in vitro*. NRP2 isoforms were strongly correlated with the presence of macrophages in both primary and metastatic human breast cancer with levels up- or downregulated within ovarian and brain metastases, respectively. High-dimensional single-cell analysis identified unique TAM subsets in both murine mammary tumors and human malignant pleural effusions (MPEs) from breast cancer patients that were distinct from NRP1-expressing TAMs. These findings highlight the importance of understanding the role of NRP2 isoforms in macrophage biology and as a potential therapeutic target in the prevention of cancer metastasis.

## Materials and Methods

### Collection of Patient Specimens

For collection of malignant and benign pleural effusions, informed consent for participation was obtained prior to effusion drainage from all patients, and no subjects were under the age of 18. The use of human tissue samples and the experiments were approved by the Institutional Review Board at the University of Pittsburgh (IRB#PRO16110093). Samples were collected as excess pathologic specimens and experiments were not performed on humans. Effusions were collected for clinically indicated drainage of symptomatic effusions, either by thoracentesis, or from a temporary or indwelling tunneled catheter. All methods were carried out in accordance with relevant guidelines and regulations. Twenty-five pleural effusions resulting from breast cancer (N = 13) or benign etiology (N = 12), most commonly due to heart failure, were included. Quantities of 350cc-1000cc were processed immediately upon collection wherein red blood cells were removed using BD Pharm Lyse (BD Biosciences) per manufacturer’s instructions, and cell pellets and acellular fluid were isolated and cryopreserved. All effusions were examined by a cytopathologist.

### Animals and Tumor Inoculation

Orthotopic murine breast cancer models were conducted using the EO771 line, a reported luminal B subtype with metastatic potential originating from a spontaneous mammary carcinoma in C57Bl/6 mice, and 4T1 cells, a highly metastatic mammary carcinoma cell line derived from a mouse mammary tumor virus (MMTV) positive BALB/c mouse which shares substantial features with human triple negative breast cancers. EO771 and 4T1 cells were obtained from the American Type Culture Collection (ATCC). For the EO771 mammary tumor model, 6-8 wk old C57Bl/6 mice (Jackson Labs) were maintained in a specific pathogen-free environment at the Animal Research Facility of the Ralph H. Johnson VA Health Care System in accordance with guidelines of the US Public Health Service Policy on Humane Care and Use of Laboratory Animals and the Institutional Animal Care and Use Committee (IACUC). 2.5x10^5^ EO771 cells were inoculated orthotopically into the 4th mammary fat pad of female C57Bl/6 mice. At a volume of 1000 mm^3^, EO771 tumors were excised for analysis. For the 4T1 tumor model, experiments were approved and executed under the guidelines of the Indiana University School of Medicine IACUC in the AAALAC accredited Laboratory Animal Research Center. To acquire tumors derived from 4T1 cells, 6-8 wk old BALB/c mice (Jackson Labs) were inoculated orthotopically with 75,000 4T1 cells into the 4th mammary fat pad of female BALB/c mice. Tumors were isolated when they reached a volume of ≥1000 mm^3^.

### Generation of RawNRP2a^KD^, RawNRP2b^KD^, and Raw.shCtl Cell Lines

The murine macrophage Raw264.7 cell line (ATCC) was stably knocked down for neuropilin-2a (NRP2a) or neuropilin-2b (NRP2b) *via* transduction with lentiviral vectors delivering isoform-specific short-hairpin RNAs as previously reported (8). Three individual shRNA constructs targeting the NRP2a or NRP2b isoforms were assessed, and cell lines with the most efficient knockdown as determined by qRT-PCR and Western blot, herein termed RawNRP2a^KD^ and RawNRP2b^KD^, underwent *in vitro* analysis. Control Raw264.7 cells expressing scrambled shRNA (Raw.shCtl) were generated by transduction and puromycin selection following the protocol from Addgene using pLKO.1 (Addgene 10879), psPAX2 (Addgene 12260) and pMD2.G (Addgene 12259) for viral particle creation (20).

### RNA Isolation and Real-Time RT-PCR

Healthy murine mammary tissues and EO771 tumors were processed to generate tissue homogenates *via* mechanical disruption in the presence of Liberase TM (Millipore Sigma) per manufacturer’s protocols. Macrophages from healthy spleen and mammary tissues or EO771 tumors were isolated *via* flow-sorting sized, LiveDead negative, CD45^+^CD11b^+^F4/80^+^ cells using a MoFlo Astrios High Speed Sorter (Beckman Coulter). Subsequently, cells were lysed in TRIzol reagent (Invitrogen) and RNA was precipitated according to manufacturer recommendations. The isolated RNA was reverse transcribed with SuperScript (Invitrogen). The product of the reverse transcription was then used in triplicates for a qRT-PCR reaction with SYBR Green Fast (ABI Inc.). The CT averages were calculated for the glyceraldehyde-3-phosphate dehydrogenase (GAPDH), NRP2a, and NRP2b primers as reported previously (8). Results are reported as the percentage of GAPDH using the formula: %GAPDH = (2−ΔCt) × 100.

### Single-Cell qRT-PCR

Automated single-cell qRT-PCR was performed on live, flow-sorted CD45^+^CD11b^+^F4/80^+^ TAMs isolated from EO771 mammary tumors using the C1 and BioMark HD systems (Fluidigm). 96 cells were loaded by the C1 system into integrated fluidics circuits (n = 6) for analysis of 96 chosen genes *via* the TaqMan Delta Gene Expression assay in the BioMark HD platform (**Supplementary Table 2** for primer details). The capture sites that did not contain single cells or were of poor quality were noted and were removed from downstream analysis. Samples and assays were loaded on the 96.96 BioMark Dynamic Array Chip for Gene Expression following manufacturer’s instructions. Briefly, 3.6 μl of 1:5 diluted cDNA was mixed with 4.4 μl of a 1:10 mixture of Fluidigm Sample Loading Regent and Taq Universal PCR Master Mix to create the real-time reaction sample mix. Equal volume of 20X TaqMan assay and Fluidigm GE Assay Loading Reagent prior were combined to generate the 10X assay mix. 5 μl of each mix was loaded on the chip inlets. BioMark qPCR was performed using the GE 96.96 Standard V.1 protocol with 40 cycles of PCR and analyzed using the auto initialized Ct thresholds for each detector. Two unique primer sets per NRP2 isoform were generated for detection of murine NRP2a (variant 1, NM_001077403.1, between coordinate 3380 and 6739) and NRP2b (variant 6, NM_001077407.1, between coordinate 3314 and 4696) for use in the Delta Gene Target Assay. Primer sets with the greatest sensitivity (NRP2a.1 and NRP2b.1) were used for further analysis of TAM subsets. Data was analyzed using Singular (Fluidigm) and SeqGeq (BD Biosciences) software packages. Relative qPCR values are reported as fold change of NRP2a high over NRP2a low expressing TAMs or NRP2b high over NRP2b low expressing TAMs. Absolute values were calculated as the percentage of GAPDH as above.

### Cytokine Measurements

To measure cytokine production, 10^6^ Raw.shCtl, RawNRP2a^KD^, or RawNRP2b^KD^ cells were incubated in 500 μl complete DMEM (10% FBS, 1% HEPES, 1% non-essential amino acids, 1% penicillin-Streptomycin) with or without 5 μg/ml LPS for 0, 1, 2, 3, 4, 8, or 24 hours. Following incubations, 50 μl culture supernatant was assayed with the cytometric bead array mouse inflammation kit (BD Biosciences) measuring IL-6, IL-10, CCL2, IFNγ, TNFα, and IL-12p70 per the manufacturer’s instructions. Data and was collected on a 4-laser BD LSR Fortessa flow cytometer and analyzed with FlowJo V10.7 (BD Biosciences). Results represent a minimum of six independent experiments.

### Phagocytosis and Lysosomal Processing Assay

For tumor cell phagocytosis, apoptosis was induced in EO771 cells using 50 μmol etoposide (Abcam) for 16 hours at 37°C, 5% CO_2_. Cells were pelleted, washed twice with PBS, and labeled with pHrodo Red succinimidyl ester (Thermo Scientific) per manufacturer’s instructions. Apoptotic EO771 cells were then co-cultured for 1 hour in triplicate at 10:1 in 96-well plates with adherent Raw.shCtl, RawNRP2a^KD^, or RawNRP2b^KD^ cells labeled with Hoechst 33342 solution at 0.1 μg/ml for 10 minutes (Thermo Scientific). Subsequently, unphagocytosed cells were removed by washing with PBS and co-cultures were imaged with the CellInsight CX7 LZR High-Content Screening Platform at 30 minutes and 4 hours and data analyzed using the HCS Studio Cell Analysis Software (Thermo Scientific). Phagocytosis and subsequent processing of pHrodo-labeled EO771 cells in the acidic environment of the lysosome induces red fluorescence with co-expression of red fluorescence with DAPI, reported by the CircSpotTotalArea metric, indicating processing of pHrodo^+^ EO771 cells.

### Tumor Cell Migration

Migration assays were performed using a 6.5 mm Transwell inserts with 8 μm pores (Corning). 4T1 and EO771 cells were serum-starved by overnight culture in RMPI or DMEM without fetal bovine serum, respectively. 50,000 serum-starved 4T1 or EO771 cells were resuspended in 100 μl serum-free media and added to the upper chamber of the Transwell insert. 600 μl of serum-free media, complete media, or conditioned media from Raw.shCtl, RawNRP2a^KD^, or RawNRP2b^KD^ stimulated for 16 hours with either media alone or 5 μg/ml LPS was added to the lower chambers. Transwells were incubated for 6 hours at 37°C 5% CO_2_. Subsequently, unmigrated cells from the upper chamber were removed with a cotton swab, then membranes were fixed in ice cold 100% methanol for 10 minutes and stained with 0.5% crystal violet in 25% methanol for 10 minutes. Membranes were extensively washed in DI water and allowed to dry. Membranes were digitally imaged at 20x using a Fisherbrand™ inverted microscope with C-mount digital camera (Fisher Scientific) and migration measured as the mean value of 4 randomly imaged fields per membrane. At least three experimental repeats were conducted.

### Flow Cytometry and Cell Sorting

Immunophenotyping was performed on fresh single-cell homogenates of mouse tissues or cryopreserved cellular fraction of pleural effusions as described previously (21–24). Detection of NRP2^Total^ (extracellular labeling) and NRP2b (intracellular labeling) was performed simultaneously along with macrophage phenotyping under standardized conditions. Data collection for spectral cytometry was conducted on all samples at the same time to eliminate batch effects. 1-5x10^6^ cells per sample were stained in Cell Staining Buffer (BioLegend) using combinations of mAbs followed by labeling with amine-reactive viability dye (LiveDead, Molecular Probes) (**Supplementary Table 1** for mAbs details). For analysis of mouse cells, surface Fc receptors were blocked by addition of FcBlock (anti-CD16/CD32; BioLegend) prior to extracellular staining. Cells were labeled extracellularly with specific mAbs then fixed, permeabilized, and labeled for intracellular expression antigens in appropriate buffers using the FoxP3 True-Nuclear Transcription Factor Buffer Set (BioLegend) per manufacturer’s instructions. Detection of NRP2b was performed using polyclonal 9080 antibody generated by R.M. Gemmill and subsequently licensed Millipore Sigma (8). NRP2b-specific 9080 was conjugated to PE using the Zenon Rabbit IgG Labeling Kit (Molecular Probes) per manufacturer’s instructions. Mouse macrophages were defined as CD45^+^CD11b^+^F4/80^+^ unless otherwise stated and human macrophages were defined as CD45^+^CD11b^+^CD14^–^CD68^+^CD66b^–^. Fluorescence minus one gating controls were used for each panel. Data was collected on a five laser Cytek Aurora spectral cytometer (Cytek Biosciences) or five laser BD LSR Fortessa (BD Biosciences). FlowJo V10.7 software was used for conventionally gated data analysis represented as the percentage or delta median fluorescence intensity (MFI), calculated as the MFI of the target staining minus the MFI of the isotype control, of the target cell population as indicated. For computational analysis, unsupervised clustering of samples was performed using rPhenograph in the Cytofkit package for R studio as previously described (21, 23, 25). rPhenograph is a graph-based partitioning method which dissects nearest-neighbor data into phenotypically coherent populations based on relatedness. Briefly, cell populations of interest were manually gated using FlowJo. Preprocessing was performed to generate expression matrix for each sample in a Flow Cytometry Standard (FCS) file. Parameters of interest were selected, and FCS files were exported and uploaded into Cytofkit package. FCS files were transformed using automatic logicle transformation (autoLgcl) and merged in to one matrix using ceil. Up to 10,000 cells per sample were clustered using rPhenograph and visualized using t-Distributed Stochastic Neighbor Embedding (tSNE). Heatmaps were generated depicting rPhenograph clusters and marker expression per cluster and per group.

### Statistical Analysis/Bioinformatics

All results were expressed as means ± standard error of the mean (SEM) unless otherwise stated. Data were analyzed using non-parametric Mann–Whitney U tests for comparisons of patient groups, paired Wilcoxon test for testing NRP2 expression between matched primary and metastatic patient tumors, and unpaired Student’s t-test for analysis of changes in *in vitro* determinations of Raw.shCtl, RawNRP2a^KD^, or RawNRP2b^KD^ cells. Two-tailed Spearman rank-order correlation tests were performed using a confidence interval of 95%. RNA sequencing and immune subset deconvolution has been previously described (26–29). Exome-capture RNA sequencing (ecRNA-seq) of 99 patient-matched primary breast tumors and metastatic tumors were collected from brain, bone, ovary and gastrointestinal tract, as previously reported (26, 27). Immune cell subset abundance was inferred from RNAseq data using the CIBERSORT deconvolution method (28, 29). Heatmaps of scqPCR and spectral cytometry data depicting normalized median values were generated with Morpheus software (Broad Institute; https://software.broadinstitute.org/morpheus) using Euclidean distance hierarchical clustering. GraphPad Prism9 (GraphPad Software) was used for statistical analysis and to generate graphics. For all hypothesis tests, a p < 0.05 was considered statistically significant.

## Results

### Expression of NRP2 and Its Isoforms Are Higher on TAMs From Murine Mammary Tumors

**Figure 1:**
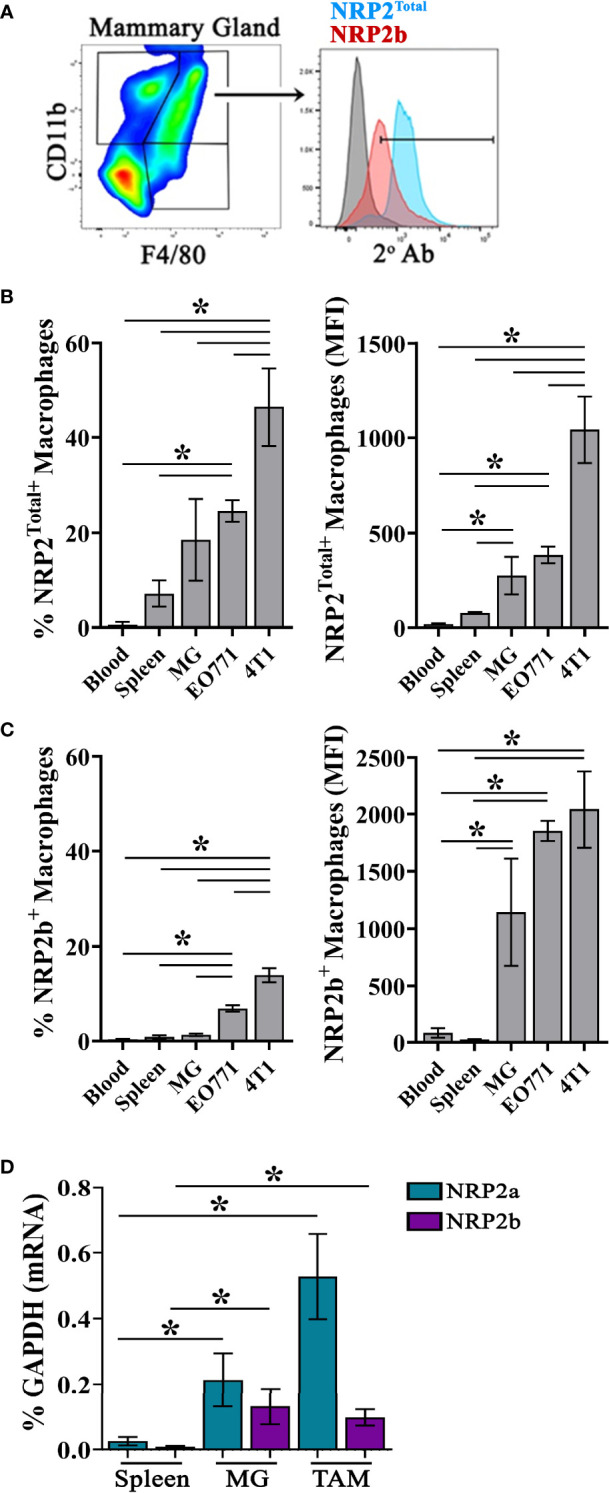
NRP2 and NRP2 isoform expression on mouse macrophages and TAMs: **(A)** Representative gating of Live, single, CD45^+^CD11b^+^F4/80^+^ macrophages from C57Bl/6 mice and subsequent expression of NRP2b and NRP2^Total^ by flow cytometry. Percent positive (left) and isotype subtracted median fluorescence intensity (MFI; right) quantitation of **(B)** NRP2^Total^ and **(C)** NRP2b expression by flow cytometry in CD45^+^CD11b^+^F4/80^+^ monocytes from the blood (N = 4), and macrophages from the spleen (n = 5) or mammary gland (N = 5; MG) of healthy mice, or orthotopic EO771 (n = 14) and 4T1 (n = 9) mammary tumors. **(D)** Transcript expression of NRP2 isoforms represented as the percent of the GAPDH gene from flow-sorted macrophages from the spleen, normal mammary gland, or EO771 tumors. *p≤0.5.

NRP2 is upregulated on macrophages following *in vitro* differentiation from progenitors and is present on TAMs within pancreatic cancers (19). In order to determine if the NRP2 isoforms are differentially expressed in TAMs versus macrophages from normal mammary tissue, we examined healthy mammary glands and mammary tumors generated with the E0771 and 4T1 cell lines in mice. Flow cytometry was performed using a polyclonal antibody we previously generated which is specific for a unique intracellular sequence near the C-terminus of NRP2b together with a validated antibody to the shared N-terminus of NRP2a and NRP2b recognizing total NRP2 (NRP2^Total^; **Figure 1A**) (8). At this time there is no commercially available antibody to detect NRP2a. Although largely absent from the minor population of CD45^+^CD11b^+^F4/80^+^ monocytes in the blood, NRP2^Total^ was detected on a subset of macrophages in the spleen (7.226%) and mammary glands (18.54%) of healthy C57Bl/6 mice (**Figure 1B**). Notably, both the percentage of NRP2^Total+^ macrophages as well as the level of NRP2^Total^ protein determined *via* MFI were significantly higher in TAMs from 4T1-derived tumors (46.43%) compared to macrophages from normal mammary glands or TAMs from EO771-derived tumors (24.58%) (**Figure 1B**). Monocytes of the blood (0.377%) and macrophages in the spleen (0.878%) expressed little NRP2b (**Figure 1C**). We observed a minor subpopulation of macrophages expressing NRP2b in the mammary glands (1.314%), yet overall NRP2b protein levels on macrophages from healthy tissues were prominently expressed suggesting that most macrophages from the mammary gland are positive for NRP2b, albeit with a much lower expression level. The percent of TAMs from EO771 and 4T1 tumors expressing NRP2b were ~5-fold (6.835%) and ~10-fold (13.99%) greater compared to macrophages in healthy mammary tissues, respectively (**Figure 1C**). Collectively, these findings suggest that NRP2a (inferred from the percent NRP2^Total^ minus NRP2b) represents the predominant isoform in tissues, although levels of NRP2b were increased modestly in TAMs from EO771 and 4T1 tumors.

We next measured transcript levels of the NRP2 isoforms within macrophages flow-sorted from the spleens and mammary glands of normal C57Bl/6 mice or from EO771 tumors. Both *NRP2a* and *NRP2b* transcripts were significantly greater in macrophages isolated from the normal mammary glands and TAMs than in macrophages from the spleen (**Figure 1D**). As observed at the protein level, *NRP2a* transcripts represented the predominant NRP2 isoform at the mRNA level. By contrast, *NRP2b* transcripts in TAMs were comparable to those in mammary gland macrophages. As NRP2b protein expression was modestly higher in TAMs from EO771 tumors (**Figure 1C**), translational regulation of NRP2 isoform expression may occur as we have previously identified (8). Together, these findings demonstrate that NRP2 isoform levels are increased on macrophages in the mammary gland and further upregulated in TAMs compared to counterparts in the blood or spleen.

### NRP2 and the NRP2b Isoform Denote Phenotypically Distinct TAMs in Murine Mammary Tumors

**Figure 2:**
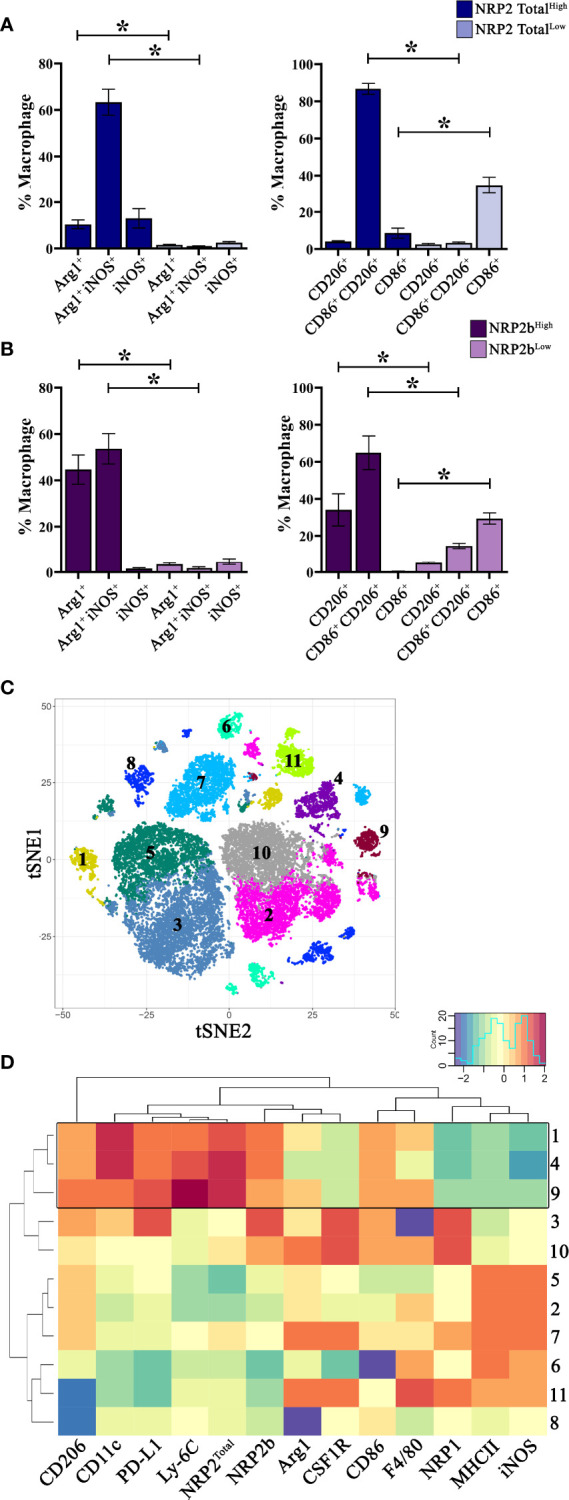
NRP2 and NRP2b expression are associated with distinct TAM phenotypes in mouse mammary tumors: Mice were orthotopically inoculated with EO771 (N = 5) or 4T1 (N = 9) mammary adenocarcinoma cells and resulting tumors were analyzed by flow cytometry comparing TAMs expressing the highest and lowest 10% of NRP2^Total^ or NRP2b. Quantitation of expression of arginase1, iNOS, CD86, and CD206 by conventionally gated flow cytometry on **(A)** NRP2^Total^ and **(B)** NRP2b expressing TAMs within EO771 tumors. Subsequently, 4T1 mammary tumors were phenotyped using a 33-color spectral cytometry. **(C)** tSNE plot illustrating CD45^+^CD11b^+^Ly-6G^–^ TAMs from 4T1 tumors analyzed *via* rPhenograph identified 11 cell subsets. **(D)** Factor heatmap depicting relative marker expression on the 11 identified TAM subsets illustrating unique phenotype of TAMs co-expressing high levels of NRP2 and NRP2b (boxed). *p≤0.5.

To examine associations between NRP expression and macrophage phenotype, we evaluated TAMs from mice bearing EO771 mammary tumors for putative markers of inflammation (CD86, iNOS) and wound-healing (CD206, Arginase-1) by flow cytometry. Utilizing conventional gating strategies, we compared TAMs expressing the highest and lowest 10% of NRP2^Total^ or NRP2b. TAMs which were high in total NRP2 (NRP2 Total^High^) expressed significantly greater levels of both arginase-1^+^ and arginase-1^+^iNOS^+^ double positive cells compared to NRP2 Total^Low^ counterparts (**Figure 2A**). NRP2 Total^High^ TAMs predominantly co-expressed both CD86^+^ and CD206^+^ whereas NRP2 Total^Low^ TAMs were almost exclusively CD86^+^ (**Figure 2A**). Similarly, TAMs enriched in NRP2b (NRP2b^High^) also possessed elevated levels of co-expressed arginase-1^+^iNOS^+^ and CD86^+^CD206^+^, but additionally had greater levels of both arginase-1^+^ and CD206^+^ alone when compared to NRP2b^Low^ TAMs (**Figure 2B**).

As NRP2^Total^ and NRP2b expression were greater in TAMs from 4T1 tumors compared to those from EO771 tumors, we hypothesized that any associated phenotypes would be more pronounced in TAMs using the 4T1 model. Subsequently, we utilized 33-color spectral cytometry to comprehensively phenotype TAMs from 4T1 mammary tumors (**Supplementary Table 1**) (21). Unsupervised clustering of CD45^+^CD11b^+^Ly-6G^–^ TAMs from 4T1 tumors resulted in 11 phenotypically distinct subsets (**Figure 2C**). Three subsets (clusters 9, 4, and 1) demonstrated higher relative expression of both total NRP2 and NRP2b. These TAM subsets possessed increased relative expression of PD-L1, CD206, CD11c, and Ly-6C with reduced levels of NRP1, MHCII, and iNOS (**Figure 2D**; full data in **Figure S1**). Expression of Ly-6C on TAM subsets with high NRP2/NRP2b levels suggests that these cells may be derived from peripheral monocytes recently recruited from the circulation (30, 31). Interestingly, although expression of NRP2^Total^ and NRP2b overlapped in TAMs, these subsets possessed markedly lower levels of NRP1 (**Figure 2D**). TAM subsets with high NRP1 co-expressed increased levels of CSF1R and decreased Ly-6C, indicating that NRP1 and NRP2 likely demark unique TAM subsets within murine mammary tumors.

### Single-Cell Transcriptional Profiling of NRP2 Isoform-Expressing TAMs From Murine Mammary Tumors Reveals Potential Functional States

To directly characterize both NRP2a- and NRP2b-expressing TAMs, we utilized the Fluidigm C1-BioMark system to conduct automated single-cell qRT-PCR on individual flow-sorted TAMs from EO771 tumors. Following evaluation for cell quality, 468 TAMs out of 576 C1-captured cells were analyzed using a panel of 96 genes selected to examine macrophage polarization, inflammation, immunosuppression, metabolism, autophagy, epigenetic regulation, as well as the neuropilin-plexin-semaphorin signaling axis (**Supplementary Table 2**). Levels of *NRP2a* and *NRP2b* were positively correlated within individual TAMs (r = 0.617, p = 7.054 x 10^-43^), whereas the presence of neither *NRP2a* nor *NRP2b* was associated with *NRP1* expression (p = 0.208; NRP2a, p = 0.661; NRP2b).

**Figure 3:**
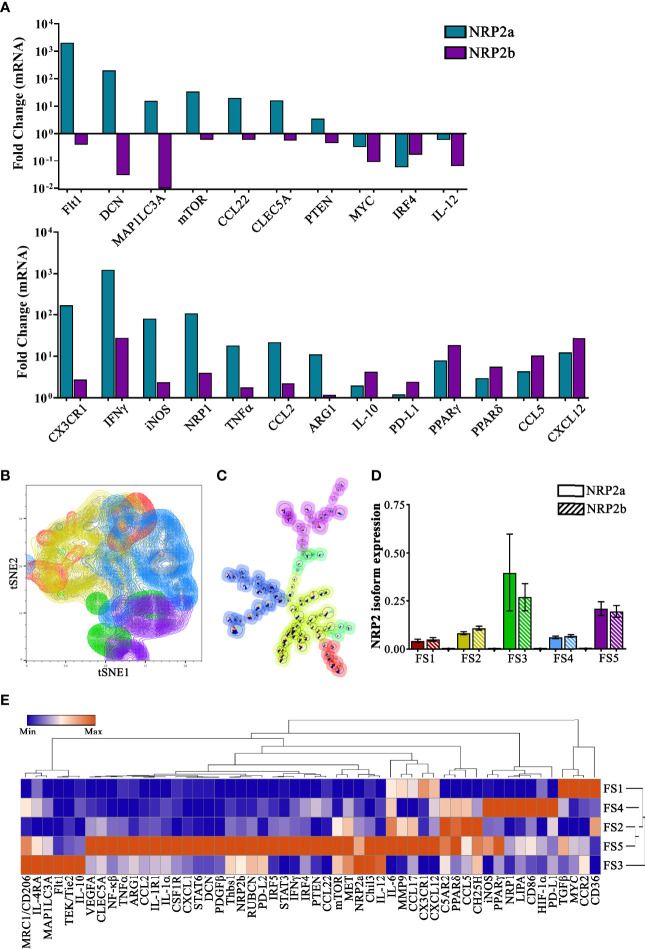
Single-cell transcriptional profiling informs the potential function of subsets of NRP2 isoform-expressing murine TAMs: TAMs were flow-sorted from EO771 mammary tumors and single-cell qPCR was performed using the Fluidigm C1-BioMark system for a 96-gene panel in 468 unique TAMs. **(A)** Transcript levels were evaluated comparing differential gene expression between TAMs with the 10% highest levels of *NRP2a* or *NRP2b* compared to TAMs containing the lowest 10% expression of NRP2 isoforms and select data illustrating differential expression, upregulation, or downregulation of factors are presented. **(B)** tSNE plots of principal component analysis of scqPCR expression delineates five subsets of TAMs with groups FS3 (green) and FS5 (purple) containing increased levels of both *NRP2a* and *NRP2b* transcripts. **(C)** FlowSOM lineage hierarchy illustrating close proximity of groups FS3 (green) and FS5 (purple) TAM subsets with higher NRP2 isoform expression. **(D)** Quantitation of *NRP2a* and *NRP2b* levels from 468 individual TAMs contained in each of the 5 subsets defined by PCA showing elevated isoform levels in groups FS3 and FS5. **(E)** Heatmap of normalized median gene expression of each of the PCA-defined subsets showing both that TAMs expressing high levels of NRP2 isoforms are phenotypically unique as well as distinct from NPR1 expressing TAMs.

To examine differential gene expression associated with NRP2 isoforms, we compared TAMs expressing the highest and lowest 10% of either *NRP2a* or *NRP2b*. Transcripts were upregulated in 85 of 92 highlighted genes by a mean of ~78-fold in NRP2a^High^ TAMs and 77 of 92 genes by a mean of ~11-fold in NRP2b^High^ TAMs (**Figure S2**). A subset of these data comparing differential gene expression between NRP2a^High^ and NRP2b^High^ populations likely reflect important differences in TAM function (**Figure 3A**). Higher levels of transcripts for cytokines/chemokines (*IFNγ*, *TNFα*, *CCL2*, *IL-10*), transcription factors (*PPARγ*, *PPARδ*, *NF-κβ*, *IRF5*, *STAT6*), as well as markers of activation, metabolism, and signaling (*MMP9*, *CH25H*, *MET*) were observed in both NRP2a^High^ and NRP2b^High^ populations (**Figure 3A** and **Figure S2**). Only *MYC*, *IRF4*, and *IL-12*, were downregulated in both NRP2a^High^ and NRP2b^High^ TAMs (**Figure 3A**). Interestingly, the hypoxia responsive gene *Flt1* (encoding VEGFR1) is expressed >5,000-fold higher in NRP2a^High^ *vs* NRP2b^High^ TAMs. In addition, *mTOR* and *PTEN* are significantly higher in NRP2a^High^ TAMs. PTEN activity underlies much of the differential functions mediated by NRP2 isoforms, and *PTEN* message was found to be >5-fold higher in NRP2a^High^ *vs* NRP2b^High^ TAMs. We have previously shown that NRP2b results in a GSK3β-dependent phosphorylation of PTEN leading to its proteosomal degradation, and concordant regulation of both PTEN transcript and protein may provide a mechanistic link between NRP2b expression and activation of AKT-related survival (9). Lastly, *PD-L1* was ~2-fold higher in NRP2b^High^ *vs* NRP2a^High^ TAMs, adding to the growing body of data suggesting that NRP2 isoforms and PD-L1 expression are related.

To better understand the association of NRPs with potential TAM subsets *in vivo*, we performed principal component analysis followed by unsupervised clustering of transcriptional profiles from individual cells. tSNE plots illustrate 5 subsets of TAMs using our 96-gene panel (**Figure 3B**). Subset relatedness was determined *via* FlowSOM with TAMs in groups 3 (FS3; green) and 5 (FS5; purple) possessing the highest expression of both NRP2 isoforms and a close hierarchical relationship (**Figures 3C, D**). TAMs within FS3, which had a greater ratio of *NRP2a* to *NRP2b*, had higher expression of multiple factors associated with alternative activation including *Chil3*, *MET*, *CD206*, *Flt1*, *IL-4RA*, *Tie2*, and *IL-10*, suggesting these may possess immunosuppressive and/or tolerogenic functionality (**Figure 3E**). Additionally, expression of the angiopoietin 1 receptor Tie2 may indicated that FS3 TAMs are involved in tumor angiogenesis (32). In contrast, TAMs in FS5 showed upregulation of numerous pro-inflammatory factors such as *NF-κβ*, *IRF5*, *PTEN*, *TNFα*, *IL-1α*, *IFNγ*, and *IL-6* as well as those regulating immunosuppression including *STAT3/STAT6*, *IRF4*, and *MMP-9*. TAMs in FS5 expressed greater *VEGFA* and *Arg1*, potentially indicating a response to hypoxic regions of the TME. Interestingly, TAMs from FS3 expressed CX_3_CR1^Low^CCR2^+^ whereas, those in FS5 were characterized by CX_3_CR1^+^ CCR2^Low^ suggesting that TAMs in these subsets may have arisen from distinct classical and non-classical monocytes, respectively (**Figure 3E**) (33). TAMs in subset FS4 were characterized by low expression of *NRP2a* and *NRP2b* but high expression of *NRP1*, and were associated with increased *HIF-1α*, *LIPA*, *PPARγ*, *PD-L1*, *iNOS*, and *CD86* expression (**Figure 3E**). Collectively, these findings provide phenotypic profiles that may inform the effects of individual and/or ratioed NRP2 isoform expression on signaling dynamics in TAMs.

### NRP2 Expression Is Associated With Macrophage Infiltration of Primary and Metastatic Breast Tumors

**Figure 4:**
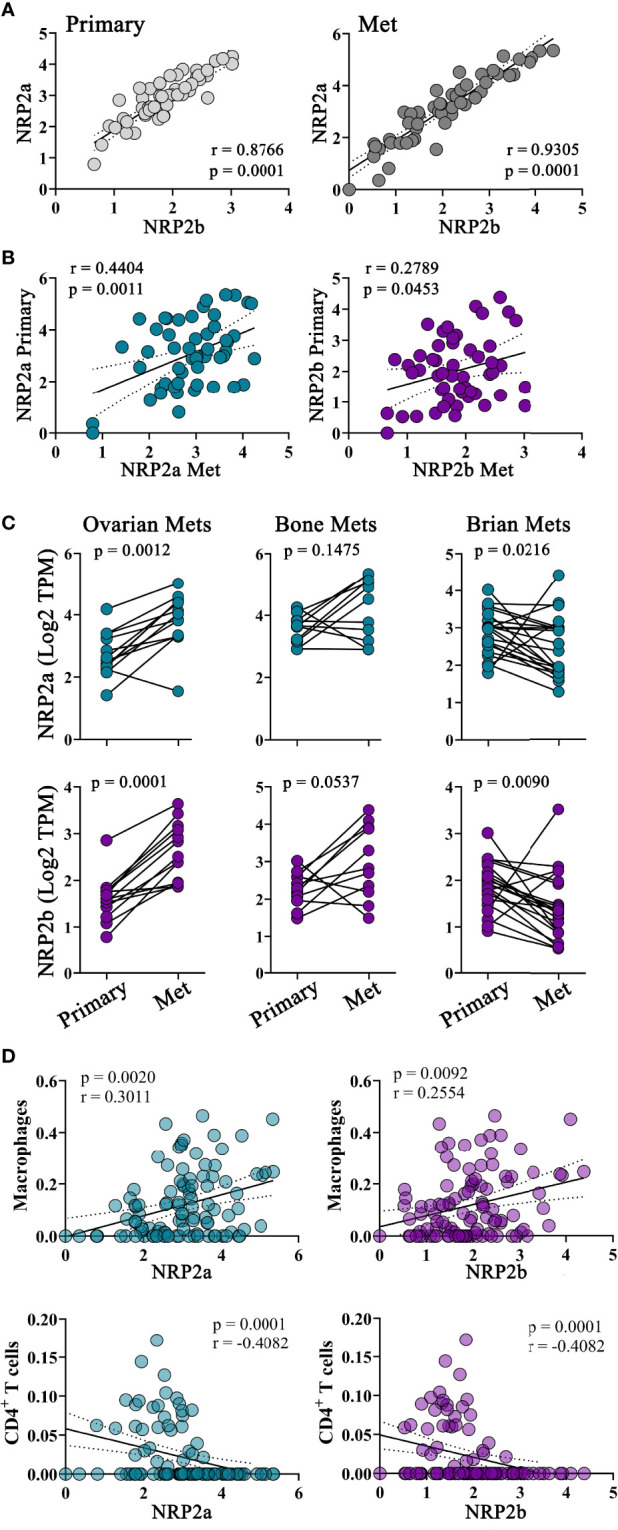
NRP2 expression is associated with increased TAM infiltration in primary and metastatic human breast cancers: To evaluate NRP2 isoform expression in a clinical setting, we determined transcript levels from a local cohort of 99 subjects where ecRNA-seq analysis was conducted on both primary breast cancers and the subsequent metastatic tumors. **(A)** *NRP2a* and *NRP2b* were strongly co-expressed among all subjects in both primary and metastatic tumors. **(B)** Expression of each isoform in primary tumors was also associated to a lesser degree within expression of the same isoform in patient matched metastatic tumors. **(C)** *NRP2a* and *NRP2b* expression was found to increase in metastasis to the ovary, decrease in metastasis to the brain, and remain relatively unchanged in bone metastasis compared to levels in patient matched paired primary tumors. **(D)** Correlations between the presence of *NRP2a* and *NRP2b* and the inferred macrophage and CD4^+^ T cell infiltrate generated *via* Cybersort deconvolution algorithm of ecRNA-seq data were determined evaluating both primary and paired metastatic tumors together.

Although NRP2 is frequently overexpressed in tumors and associated with poor clinical outcomes, the role of the NRP2 isoforms and their impact on macrophages in the TME remains undefined (8, 34–39). We examined specimens from 99 breast cancer patients where both primary tumors and metastases underwent ecRNA-seq. Similar to our mouse mammary cancer models, the *NRP2a* and *NRP2b* isoforms were highly co-expressed in both primary and metastatic tumors (**Figure 4A**). Levels of *NRP2a* or *NRP2b* were also positively correlated with themselves within a patient’s paired primary and metastatic tumor (**Figure 4B**). Compared to levels in primary tumors, NRP2 isoform expression was increased in patient-matched ovarian metastases, decreased in brain metastases, and was unchanged in bone metastases, suggesting that there may be a relationship between metastatic location and NRP2 expression (**Figure 4C**). Using CIBERSORT deconvolution to approximate the presence of leukocytes within primary and metastatic tumors from bulk ecRNA-seq data, we identified a significant positive correlation between macrophages and both *NRP2a* and *NRP2b* expression in combined primary and metastatic tumors (**Figure 4D**). Significant correlations were also present for the association of *NRP2a* or *NRP2b* and macrophages within either primary tumor or metastatic tumor alone. Interestingly, both NRP2 isoforms were inversely correlated with CD4^+^ T cells in combined primary and metastatic tumors but no association was observed with CD8^+^ T cells (**Figure 4D**, data not shown). Collectively, these data suggest that differential regulation of NRP2 isoforms occurs among sites of metastasis, and that NRP2 expression is associated macrophage infiltration of the TME.

### NRP2b-Expressing Macrophages Are Enriched Within Malignant Pleural Effusions From Patients With Breast Cancer

**Figure 5:**
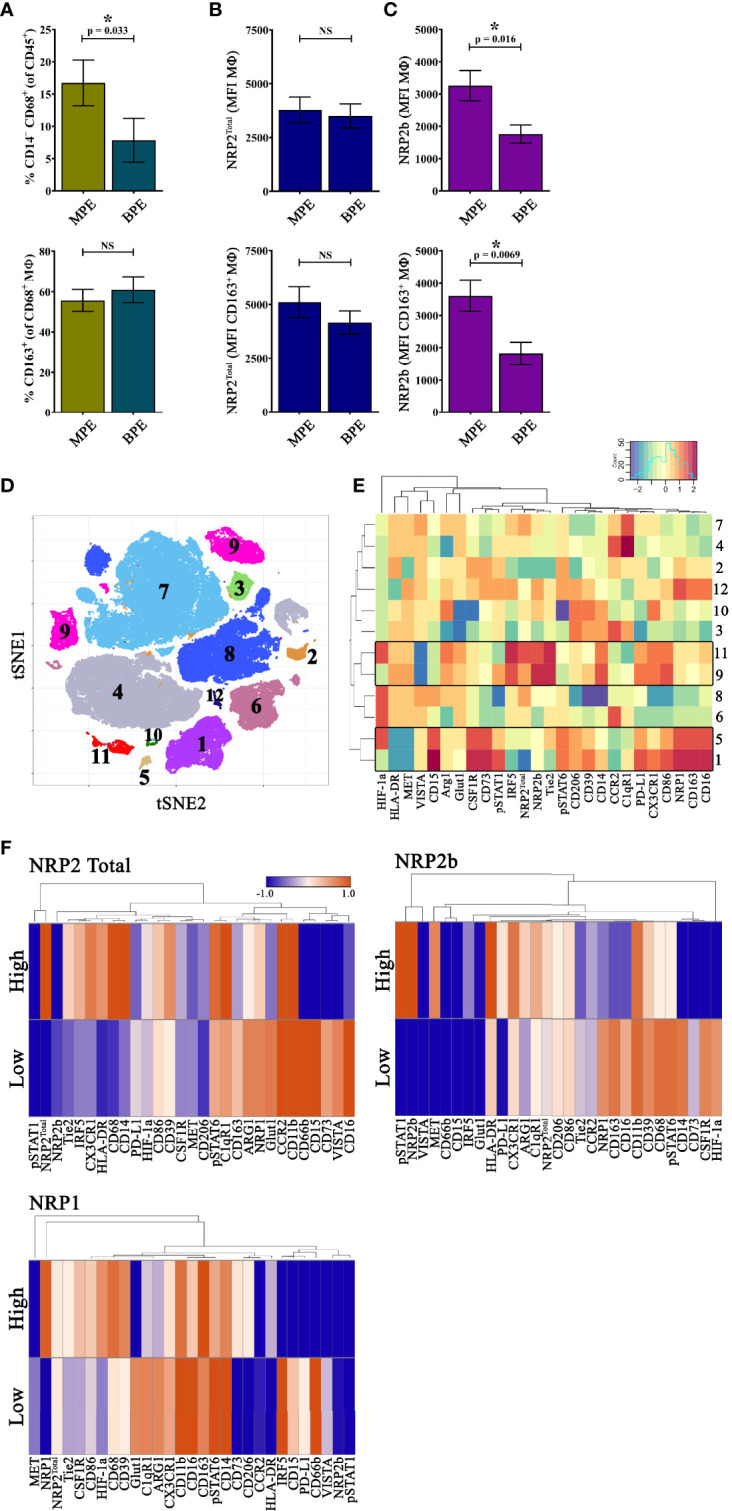
NRP2b-expressing TAMs are enriched in MPEs secondary to human breast cancer: To evaluate macrophage expression of NRP2^Total^ and NRP2b in the human metastatic setting, we evaluated MPEs secondary to breast cancer (N = 13) and BPEs resulting from heart failure (N = 12) using 32-color spectral cytometry. **(A)** The percentage of live CD45^+^CD11b^+^CD14^–^CD68^+^CD66b^–^ macrophages from CD45^+^ leukocytes (top) and the CD163^+^ fraction of alternatively activated macrophages from total macrophages (bottom) from MPEs and BPEs showing increased total, but not CD163^+^ macrophages in MPEs. Expression of **(B)** NRP2^Total^ and **(C)** NRP2b on all macrophages (top) or CD163^+^ macrophages (bottom) from MPE and BPE showing increased NRP2b^+^ macrophages in MPEs. **(D)** tSNE of rPhenograph analysis of all macrophages within MPEs depicting 12 subsets based on spectral panel. **(E)** Heatmap of relative factor expression on macrophages per subsets reveals distinct phenotype of NRP2^Total^/NRP2b and NRP1 expressing macrophages. **(F)** Heatmaps illustrating differential expression of normalized median values of phenotypic markers among macrophages expressing the highest and lowest 10% NRP2^Total^, NRP2b, or NRP1. *p≤0.5.

To further examine macrophages in a human metastatic breast cancer environment, we utilized 32-color spectral flow cytometry (**Supplementary Table 1**) to evaluate the cellular fraction from malignant pleural effusions (MPEs) secondary to breast cancer and benign pleural effusions (BPE) as non-malignant controls. Using conventional gating, the percentage of macrophages (live CD45^+^CD11b^+^CD14^–^CD68^+^CD66b^–^) but not alternatively activated CD163^+^ macrophages from total CD45^+^ leukocytes was significantly higher in MPEs than in BPEs (**Figure 5A**). Total macrophages and the CD163^+^ subset within MPEs did not express more NRP2^Total^ than those in BPEs, but did possess greater levels of NRP2b, indicating that the NRP2b isoform represents a greater proportion of NRP2^Total^ levels within MPEs (**Figures 5B, C**). These findings suggest that NRP2b is upregulated on macrophages in the unique metastatic environment of MPEs and that NRP2b expression is not restricted to alternatively activated CD163^+^ macrophages.

We then performed unbiased computational analysis on macrophages from MPEs to identify biologically relevant populations. Here, distinct subsets expressing high levels of NRP2^Total^ and NRP2b (clusters 9 and 11) or NRP1 (clusters 1 and 5) were identified (**Figures 5D, E**). Macrophage subsets expressing high NRP2^Total^ and NRP2b had elevated Tie2, IRF5, PD-L1, CD86, CX3CR1, and CD14, with decreased VISTA, whereas macrophages with high NRP1 had increased CD163, CSF1R, CD73, CD15, and CD16, with reduced HLA-DR and MET (**Figure 5E**). As observed in mouse mammary tumors, macrophage subsets which expressed high levels of NRP2^Total^ and NRP2b did not express significant NRP1, suggesting that NRP1 and NRP2 are associated with discrete macrophage populations in human breast cancer MPEs.

To identify phenotypic differences associated with NRP expression across all macrophages from within breast cancer MPEs, we compared subsets which expressed the highest or lowest 10% of NRP2^Total^, NRP2b, or NRP1, generating heatmaps depicting normalized median expression of phenotypic markers (**Figure 5F**). Macrophage subsets expressing high NRP2^Total^ had increased Tie2, IRF5, CX3CR1, HLA-DR, and CD68 and decreased CD73, VISTA, and CD16 compared to macrophages with low NRP2^Total^ (**Figure 5F**). High NRP2b was associated with elevated phosphorylated STAT1, MET, PD-L1, Arginase1, and HLA-DR and lower levels of NRP1, HIF-1α, CD163, and CSF1R, suggesting that within MPEs these cells possess an inflammatory phenotype (**Figure 5F**). Macrophages with high NRP1 differentially upregulated HIF-1α, CD206, CD73, Tie2, and CSF1R while downregulating Glut1, C1qR1, Arginase1, and IRF5 (**Figure 5F**). Elevated NRP2^Total^ or NRP2b was associated with increased pSTAT6/IRF5 and pSTAT1/MET, respectively, suggesting that the stochiometric ratio of NRP2 isoforms may be associated with unique environmental responses. NRP1 expression was associated with upregulation of HIF-1α and CD39/CD73 with reduced IRF5 suggesting recruitment to hypoxic regions and immunosuppressive function as has been previously reported (12). Macrophages with high NPR2b expression had dramatically reduced levels of NRP1, whereas those with high NRP2^Total^ had only modestly lower NRP1, suggesting co-expression of the more closely related NRP2a isoform with NRP1 with potential overlap in function.

### NRP2 Isoforms Regulate Cytokine Production and Phagocytosis in Macrophages

**Figure 6:**
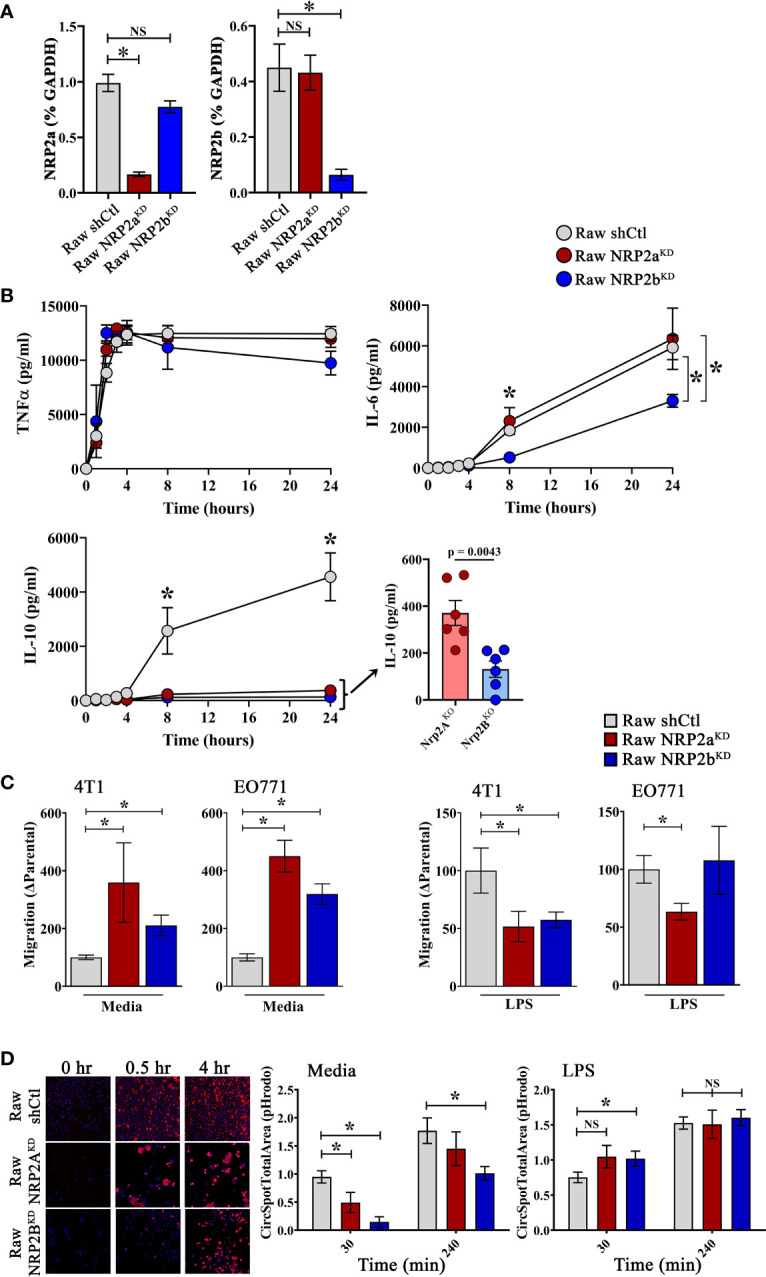
NRP2 Isoform-specific regulation of macrophage function: The Raw264.7 murine macrophage cell line was depleted of NRP2a or NRP2b *via* lentiviral transduction delivering isoform-specific shRNA to generate stable RawNRP2a^KD^ and RawNRP2b^KD^ lines or scrambled shRNA for isoform intact control cells (Raw.shCtl). **(A)** Presence of NRP2a or NPR2b measured by qRT-PCR shown as percent GAPDH and normalized to Raw.shCtl expression. **(B)** Soluble cytokines (TNFα, IL-10, IL-6) were measured *via* cytometric bead array in cell supernatants following exposure to LPS for 0 to 24h showing decreased IL-10 production from both isoform knockdowns and reduced IL-6 production from RawNRP2b^KD^ cells post-stimulation (N = ≥6 independent experiments). **(C)** Migration of serum-starved 4T1 and EO771 cells in response to conditioned media from Raw.shCtl, RawNRP2a^KD^, or RawNRP2b^KD^ cells with or without LPS stimulation was measured *via* transwell assay. **(D)** To measure degradation of phagocytosed cargo, pH-sensitive pHrodo red-labeled, etoposide-killed EO771 cells were co-cultured with Hoechst-labeled macrophages and fluorescence determined at 30 min and 4h by confocal microscopy showing reduced phagocytic uptake and/or clearance in RawNRP2a^KD^ and RawNRP2b^KD^ cells. ns stands for non-significant. *p≤0.5.

While differentially expressed in tumors and healthy tissues, the functional implications of NRP2 isoform expression in macrophages is unknown. Therefore, we generated isoform-specific shRNA knockdowns in mouse macrophage Raw264.7 cells, resulting in 83.2% and 85.7% reduction in *NRP2a* and *NRP2b* mRNA compared to scramble shRNA engineered control cells, respectively (**Figure 6A**). To assess the role of NRP2 isoforms in response to a well characterized, pro-inflammatory stimulant, cells were examined in the presence or absence of LPS. Raw.shCtl, RawNRP2a^KD^, and RawNRP2b^KD^ cells were cultured in the presence of LPS for up to 24 hours and soluble cytokines/chemokines were measured in cell-free supernatant. NRP2 isoform expression did not affect production of TNFα, CCL2, IFNγ, or IL-12p70 (**Figure 6B**, and data not shown). However, knockdown of either NRP2a or NRP2b resulted in near complete ablation of IL-10 production compared to controls (**Figure 6B**, insert). Interestingly, production of IL-6 did not differ in RawNRP2a^KD^ cells (6349 ± 1510 pg/ml) compared to Raw.shCtl (5919 ± 598 pg/ml) but was reduced by roughly half in RawNRP2b^KD^ (3301 ± 315 pg/ml) (**Figure 6B**). No differences in proliferation or death were detected between cell lines by MTT assay and LiveDead exclusion *via* flow cytometry, respectively.

Next, using conditioned media from Raw.shCtl, RawNRP2a^KD^, or RawNRP2b^KD^ cells after overnight incubation with or without LPS, we performed transwell migration assays with the mouse mammary carcinoma lines EO771 and 4T1. Exposure to conditioned media from both RawNRP2a^KD^ and RawNRP2b^KD^ cells significantly increased 4T1 and EO771 cell migration compared over levels from Raw.shCtl media (**Figure 6C**). In contrast, conditioned media from LPS-stimulated RawNRP2a^KD^ and RawNRP2b^KD^ significantly reduced migration of 4T1 cells compared to conditioned media from LPS-stimulated control cells. However, with EO771 cells, only conditioned medium from RawNRP2a^KD^ cells had this effect.

We next examined the impact of NRP2 isoforms on the ability of macrophages to phagocytose and process tumor cells. Apoptosis was induced by treatment of EO771 cells with etoposide overnight prior to labeling with the pH-sensitive fluorescent dye pHrodo Red and co-culturing with DAPI labeled macrophages. Knockdown of the NRP2a and NRP2b isoform reduced phagocytosis and/or lysosomal processing of apoptotic pHrodo^+^ EO771 cells by ~48% and ~86%, respectively, after 30 minutes and by ~18% and ~43%, respectively, after 4 hours co-incubation, indicating that NRP2b regulated phagolytic processing to a greater extent than NRP2a (**Figure 6D**). LPS has been shown to enhance macrophage phagocytosis, and pre-treatment of macrophages with LPS for 24 hours prior to co-culture augmented function at 30 minutes and restored full phagolytic function in both RawNRP2a^KD^ and RawNRP2b^KD^ cells at 4 hours (**Figure 6D**). These findings indicate that both NRP2 isoforms possess common as well as unique roles in regulating specific cytokine production and phagocytic uptake and/or lysosomal processing in macrophages.

## Discussion

Neuropilins regulate function in T cells, dendritic cells, and macrophages, but the role of the NRP2 isoforms in immunity has yet to be examined (19, 40, 41). Given our findings that NRP2a and NRP2b regulate distinct signaling pathways resulting in opposing biologic function in lung cancer cells, we hypothesized that these receptor isoforms may also associate with unique macrophage subsets in tumors (8, 9). Here we demonstrate the presence of NRP2a and NRP2b in macrophages, the co-enrichment of both isoforms in TAMs within murine and human breast cancer and speculate that NRP2 and NRP1 denote unique TAM subsets. We observed a significant association between macrophages and both NRP2a and NRP2b in human primary and metastatic mammary tumors, with levels of the NRP2 isoforms varying dramatically between metastatic sites. Employing single-cell qPCR, we found a strong positive correlation between NRP2a and NRP2b transcripts within individual TAMs, but not with the related NRP1. Similarly, TAM subsets within murine mammary tumors or MPEs from breast cancer patients expressed predominantly NRP1 or NPR2^Total^/NRP2b by flow cytometry, suggesting that expression of NRP1 or NRP2^Total^/NRP2b on TAMs is associated with non-redundant functions in the breast cancer TME. NRP expression is responsive to environmental signals with upregulation of NRP2^Total^ detected on TAMs within the TME and on alveolar macrophages following LPS administration (18, 19). Notably, metabolic stressors present in the TME including hypoxia and nutrient deprivation results in the lysosomal degradation of NRP1, but not NRP2 on endothelial cells and carcinoma cells *via* autophagy (42). This phenomenon may occur in TAMs, with NRP2-expressing cells residing within an inhospitable TME and NRP1 expressing TAMs located in regions with supportive conditions or having more recently emigrated to the tumor. Additionally, we have previously shown that TGFβ signaling preferentially induces expression of NRP2b on non-small cell lung cancer cell lines (8, 43). Interestingly, NRP2b is upregulated on macrophages within MPEs and modestly enriched in TAMs within murine mammary tumors and may either induce or result from macrophage polarization in response to microenvironmental exposures within tumors. Collectively, these findings suggest that regulation of NRP1 and the NRP2 isoforms, along with their potential function, may vary due to cellular ontogeny, exposure to stimuli, or location within the TME.

Our findings highlight the unique nature of the NRP2 isoforms, regulating both distinct as well as shared functions in macrophages. Most strikingly, both isoforms were necessary for production of IL-10, but only NRP2b was required for full production of IL-6 following LPS exposure. Similarly, both isoforms were involved in the phagocytosis and lysosomal processing of apoptotic tumor cells, but loss of NRP2b on macrophages resulted in more significant and prolonged inhibition of function. These findings are consistent with previous reports that NRP2^Total^-knockout CD11b^+^ myeloid cells isolated from pancreatic tumors in mice displayed reduced *IL-10* mRNA production and delayed phagosomal processing compared to NRP2^Total^-intact counterparts (19). Furthermore, NRP1, NRP2a, and NRP2b may homodimerize or heterodimerize, transmitting inhibitory or stimulatory signals based on the composition of the receptor kinases recruited to the C-terminus in response to the unique extracellular ligands bound. We found that NRP2a was co-expressed with NRP1 to a greater extent than with NRP2b at the protein and transcript levels and NRP1 shares greater structural similarity with NRP2a than NRP2b, with 54% and 11% amino acid sequence homology in the C-terminus of the cytoplasmic domain, respectively. These findings suggest potential related and/or common functionality between NRP1 and NRP2a and illustrate the unique role of NRP2b.

Upon LPS stimulation, IL-10 is upregulated by a delayed autocrine/paracrine loop subsequent to the induction of type I interferons and propagated *via* TRIF-mediated activation of the Sp1 promoter and p38MAPK/STAT3 signaling axis (44–47). Activation of the PI3K-Akt pathway and inhibition of PTEN enhances IL-10 production in macrophages following LPS challenge (48, 49). Through RTKs and GSK3β, NRP2b promotes Akt and inhibits PTEN. Accordingly, NRP2b^High^ TAMs in murine mammary tumors had increased *IL-10* levels compared to TAMs with low NRP2b. In contrast, NPR2 isoform-specific signaling effects may result from interaction with endocytosis/endosomal trafficking factors, such as GIPC1 and APPL1 in the case of NRP2a, consistent with NRP-mediated trafficking of VEGFR2 and EGFR (50–52). Additionally, insight into this phenomenon may be gleaned from SHP-1. SHP-1, through a complex of Src and Pyk2 tyrosine kinases, serves as a positive regulator of LPS-induced IL-10 and IL-6 in macrophages *via* distinct pathways involving C/EBPb and NF-κβ (53, 54). Splenic macrophages from SHP-1-knockout mice secrete significantly less IL-10 and IL-6 but equivalent or elevated TNFα in response to LPS compared to intact counterparts (53). Yet, no interaction between SHP-1 and NRPs has been reported. Interestingly, GSK3β inhibition, which would putatively impede NRP2b-mediated responses, suppresses NF-κβ and uniformly decreased TNFα, IL-6, and IL-10 production in response to LPS, suggesting NRP2b-specific cytokine regulation does not act through acts through GSK3β (55, 56). Collectively, the dramatic effect upon IL-10 production and processing of apoptotic cells indicates a strong dependency on global NRP2 expression which may serve as a necessary co-receptor, regulate cytoskeletal rearrangement, and endosomal trafficking required for TLR signaling. Further definition of signaling pathways modulated by both homodimers and heterodimers of the NRP2 isoforms may provide targets for therapeutic disruption of pro- and anti-inflammatory responses in macrophages.

Following recruitment to avascularized, hypoxic regions of mammary tumors TAMs support angiogenesis facilitating tumor growth and metastasis (57, 58). Casazza et al. demonstrated that a subset of TAMs are recruited to hypoxic regions by tumor-derived Sema3A whereby NRP1 transactivates VEGFR1 (12). Once positioned within the hypoxic niche, these TAMs downregulate NRP1 and secrete pro-angiogenic factors such as VEGF, Sema3A, MMP2, and MMP9 (59, 60). Notably, *Flt1* (encoding VEGFR1) was the most highly differentially upregulated transcript in murine NRP2a^High^ TAMs, but *Flt1* was downregulated in NPR2b^High^ TAMs, implying that VEGFA signaling in NRP2a^High^ TAMs likely occurs through VEGFR1, but not in NRP2b^High^ TAMs where it likely occurs through the alternative VEGF receptor, NRP2, with profoundly distinct consequences. NRP2a^High^ TAMs had increased levels of *MMP9* (30.5-fold), *Tie-2* (20-fold), *VEGFA* (14.5-fold), and *HIF-1α* (10.6-fold) transcripts compared to NRP2a^Low^ TAMs, suggesting that NRP2a may either identify a unique subset of TAMs capable of responding to tumor hypoxia, or alternatively, that factors within avascularized regions may selectively induce NRP2a expression. By contrast, the unique functions of the NRP2b isoform may themselves polarize specialized TAM subsets. On TAMs within murine mammary tumors, NRP2b was associated with increased markers of autophagy (MAPILC3A, DCN) as well as immunosuppression (PD-L1). Collectively, our studies illustrate the dynamic nature of NRP2 isoform expression in breast cancer and suggest that targeting NPR2 isoform-specific signaling pathways or NRP2-isoform expressing TAM subsets may be leveraged for macrophage reprograming or TAM-targeted immunotherapies.

## Data Availability Statement

The original contributions presented in the study are included in the article/**Supplementary Material**, further inquiries can be directed to the corresponding author.

## Ethics Statement

The studies involving human participants were reviewed and approved by The use of human tissue samples and the experiments were approved by the Institutional Review Board at the University of Pittsburgh (IRB#PRO16110093). Samples were collected as excess pathologic specimens and experiments were not performed on humans. The patients/participants provided their written informed consent to participate in this study. The animal studies were reviewed and approved by the Institutional Animal Care and Use Committees of the Ralph H. Johnson Veterans Affairs Medical Center and the Indiana University School of Medicine.

## Author Contributions

RD, KEJ, AAP, SHE, CN, ZC, Y-NG, KD, FC, ESY, and ACS collected specimens and performed experiments. RD, ACL, ESY, JDL, AVL, SO, MTL, RG, and AS analyzed and interpreted data. RD, AVL, SO, MTL, RMG, and ACS designed studies. All authors contributed to the article and approved the submitted version.

## Funding

This work was supported by awards from the Susan G. Komen Foundation (CCR15329745), U.S. Department of Defense (W81XWH1910650), Pennsylvania Department of Health (PA CURE), and American Lung Association/Thoracic Surgery Foundation to AS. RD was supported by funding from a Department of Veteran’s Affairs Career Development Award (CX001771-01A2) and the University of Pittsburgh’s Dean Faculty Advancement Award. EY was supported by the NCI of the NIH under R03 CA245774. ML was supported by the NCI of the NIH under awards R01CA181450 and R01CA206012 as well as ITTC/UPMCE. RD and AS were further supported by funding from the Department of Cardiothoracic Surgery.

## Conflict of Interest

Michael T. Lotze is the Chief Cellular Therapy Officer of Nurix Therapeutics, Inc.

The remaining authors have no conflicts of interest to declare. The authors declare that the research was conducted in the absence of any commercial or financial relationships that could be construed as a potential conflict of interest.

## Publisher’s Note

All claims expressed in this article are solely those of the authors and do not necessarily represent those of their affiliated organizations, or those of the publisher, the editors and the reviewers. Any product that may be evaluated in this article, or claim that may be made by its manufacturer, is not guaranteed or endorsed by the publisher.

## Supplementary Material

Supplementary Figure S1 Expanded phenotyping of TAMs from 4T1 mammary tumors: 4T1 mammary tumors were analyzed by 33-color spectral cytometry using an antibody panel including markers for broad immunophenotyping and myeloid cell function. Unbiased clustering was performed on CD45+CD11b+Ly-6G– TAMs using rPhenograph identifying 11 distinct cell subsets illustrated via tSNE (**Figure 2C**). Shown are an expanded phenotyping illustrating that TAM subsets with high level NRP2Total and NRP2b co-expression possessed increased CD4, CD8, CD206, CD49b, CD11c, Ly-6C, PD-L1, CD25, CD127 and the microglial marker TMEM119 with reduced levels of NRP1, CD24, CSF1R, MHCII, Siglec-F, iNOS and CD103.

Supplementary Figure S2 Complete listing of differentially expressed transcripts detected by single-cell qPCR from high versus low NRP2 isoform-expressing murine TAMs: Live, single, CD45+CD11b+F4/80+ TAMs were flow-sorted from orthotopic EO771 mammary tumors in C57Bl/6 mice and single-cell qPCR was performed using the Fluidigm C1-BioMark system for a 96-gene panel examining the NRP/Sema/Plexin signaling axis, trophic and inflammatory macrophage function, autophagy, metabolism, and cytokine/chemokine profiles in 468 unique TAMs. Transcript levels were evaluated comparing differential gene expression between TAMs with the 10% highest levels of **(A)** NRP2a or **(B)** NRP2b compared to TAMs containing the lowest 10% expression of NRP2 isoforms.

Supplementary Table 1 Antibody panels used for spectral flow cytometry of mouse and human myeloid cells.

Supplementary Table 2 Target and primer details for single-cell qPCR of murine TAMs.
