# Supplementary figures and images for: Isoforms of Neuropilin-2 Denote Unique Tumor-Associated Macrophages in Breast Cancer
Source: Front Immunol. 2022 Apr 27;13:830169. doi: 10.3389/fimmu.2022.830169 (PMC9149656; doi:10.3389/fimmu.2022.830169)

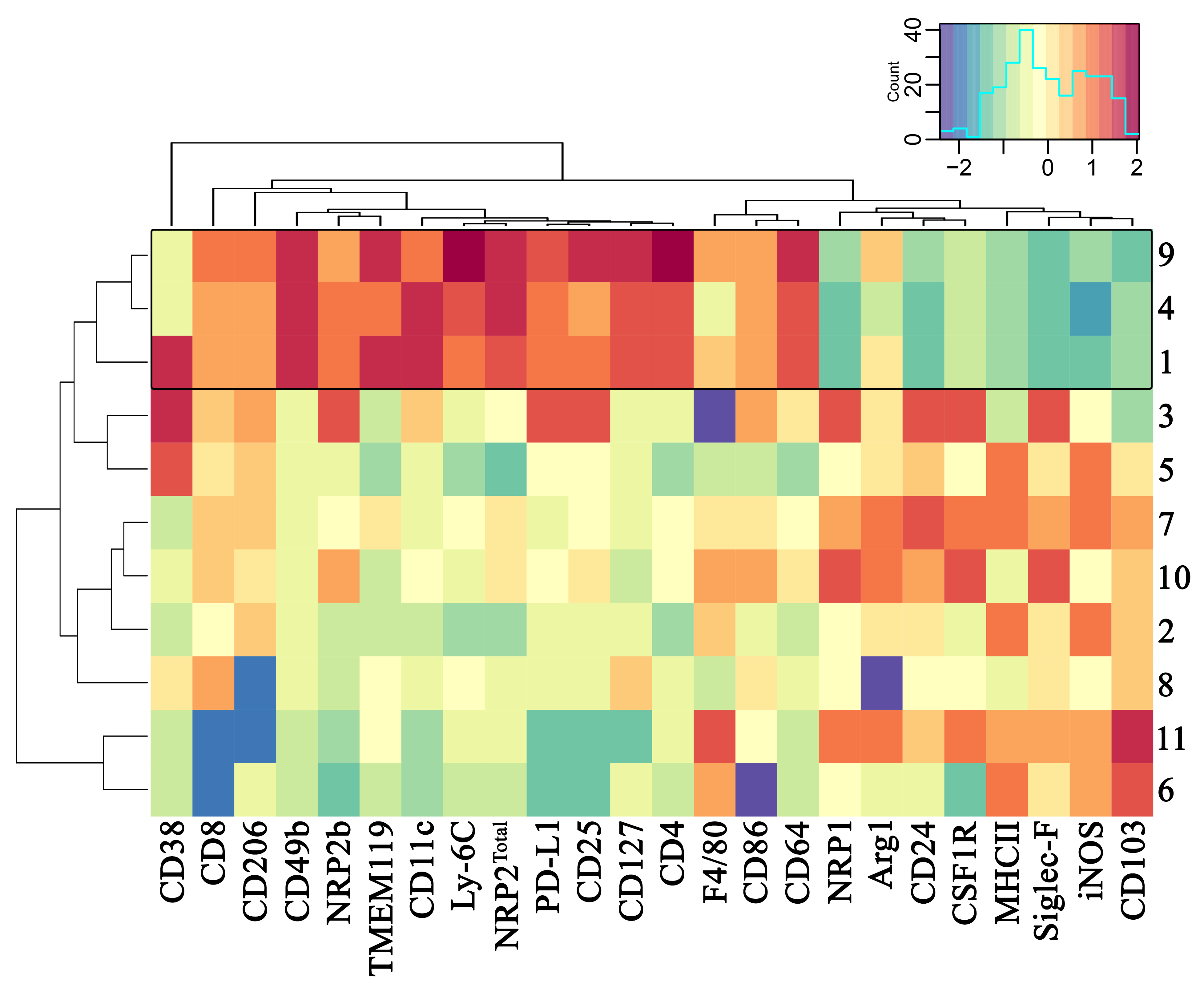

Supplement: Supplementary Figure S1 — Expanded phenotyping of TAMs from 4T1 mammary tumors: 4T1 mammary tumors were analyzed by 33-color spectral cytometry using an antibody panel including markers for broad immunophenotyping and myeloid cell function. Unbiased clustering was performed on CD45+CD11b+Ly-6G– TAMs using rPhenograph identifying 11 distinct cell subsets illustrated via tSNE ( Figure 2C ). Shown are an expanded phenotyping illustrating that TAM subsets with high level NRP2Total and NRP2b co-expression possessed increased CD4, CD8, CD206, CD49b, CD11c, Ly-6C, PD-L1, CD25, CD127 and the microglial marker TMEM119 with reduced levels of NRP1, CD24, CSF1R, MHCII, Siglec-F, iNOS and CD103. [file Image_1.jpeg]
